# Sex differences in learning and performing the Go/NoGo tasks

**DOI:** 10.1186/s13293-023-00504-z

**Published:** 2023-05-03

**Authors:** Qianwen Zhang, Mingxi Li, Zhiru Wang, Fujun Chen

**Affiliations:** 1 Bio-X Institutes, Key Laboratory for the Genetics of Developmental and Neuropsychiatric Disorders, Ministry of Education, Shanghai Jiao Tong University, Shanghai, China; 2 Exercise, Health and Technology Centre, Department of Physical Education, Shanghai Jiao Tong University, Shanghai, China; 3 The Institute of Brain Functional Genomics, East China Normal University, Shanghai, China

**Keywords:** Learning, Sex, Go/NoGo, Habit, Behavior, Performance, Strategy

## Abstract

**Background:**

The quality of learning and post-learning performances is critical for daily life. The behavioral flexibility is equally important for adapting the changing circumstances. The learning process requires repeated practices, which enhances prompt and proper behavioral responses, in turn, which promotes habits formation as well. Despite the well-documented sex differences in learning and performances, contradictory results were reported. A possible cause might be a systematic analysis due to specific research interests, regardless of the continuity of natural acquisition process. Here, we investigate the potential sex differences in learning, performances and adjustments of habited behaviors with regular and reversal Go/NoGo tasks.

**Methods:**

Both male and female Sprague-Dawley rats were used in this study. All rats were trained for a regular rodent Go/NoGo task and a subset of rats were trained for a reversal rodent Go/NoGo task, both with strict elimination criteria. The behavioral performance data were stored in PC for off-line analysis. Multiple behavioral indices were analyzed for both passed and retired rats.

**Results:**

The ability of learning the regular the reversal Go/NoGo tasks was similar for both male and female rats, however, the female rats took longer time to master the task principles in later stages for both tasks. In the regular Go/NoGo task, the female rats spent more time on completing the trial in performance optimization phases, which implied female rats were more cautious than male rats. Along with the progression of training, both male and female rats developed Go-preference strategies to perform the regular Go/NoGo task, which induced failure to meet the setting success criteria. The retired male rats exhibited shorter RTs and MTs than the retired female rats after developing Go-preference. Moreover, the time needed to complete the Go trials was significantly prolonged for male rats in the reversal Go/NoGo task.

**Conclusions:**

Overall, we conclude that distinctive strategies were employed in performing Go/NoGo tasks for both male and female rats. Male rats required less time to stabilize the performance in behavioral optimization phase. In addition, male rats were more accurate in estimating time elapsing. In contrast, female rats took more cautious considerations in performing the task, through which minimal influences were manifested in the reversal version of task.

## Highlights

- The general learning ability was similar for both male and female rats in learning the regular and reversal Go/NoGo tasks.
- The female rats took longer time to stabilize their performance in behavioral optimization phase.
- The female rats needed more time to complete the Go trials in the behavioral optimization phase, which implied the female rats were more cautious than male rats.
- Global consideration and Go-preference strategies were observed in performing the regular Go/NoGo task.
- Upon changing habited behaviors, the male rats were affected more significantly.

## Introduction

The prevalence of various neuropsychiatric disorders is significantly different between males and females [1, 2]. Moreover, the symptom severity is sex-related as well. However, the practical knowledge used by clinicians is originated from studies mainly with males [3], which may potentially bias the practices for diagnosis, treatment and prevention of disease. Therefore, researches with balanced sexes are urgently needed and should be promoted, other than to be considered as a specialized area of interest.

Among various brain functions, learning is a vital ability of adaptation to the dynamic circumstances based on previous experiences, which is pivotal for survival. Learned behaviors are distinct from innate behaviors, that the latter requires little prior experiences and exhibits sex-specific traits to certain extent. However, sex-related differences extensively exist in various learning processes [4–6]. Although the learned behaviors are shaped by evolutionary factors [7–9], varied inputs to the shared neural circuits with innate behaviors [10–12] and hormonal secretions [13, 14], etc., the underlying mechanisms remain elusive.

Prompt and accurate responses are critical in operant tasks performance. A significant body of literature indicated that females outperform males in the tasks requiring avoidance of aversive stimulation [15–18]. When the reward-driven paradigms were used, the males performed better than the females [19, 20]. However, contradictory results were reported with increased task complexity and/or difficulty levels [21, 22]. The existing evidences indicate that the sex differences are likely to be task-specific. Therefore, more in-depth analyses are required in order to further elucidate the potential causes.

Certain actions tend to be executed automatically without cognitive awareness after a great number of repetition, which evolves to habitual actions [23, 24]. When circumstances change, the behavioral flexibility is impaired to certain degree by this kind of rigid actions. Hence, the importance of revaluation for the behavioral consequences becomes pronounced under this scenario [25, 26]. Unfortunately, even in the studies including both male and female subjects, little findings were reported regarding sex differences [26–28].

Research designs with imbalanced sex subjects, from which biased conclusions might be drawn, have attracted more attention to the scientific community [29–32]. In addition, various diseases displayed sex-related prevalence and symptom severity [2, 33–37]. Therefore, the urgency of including both sexes in any research becomes prominent. In order to study the influences of sex on learning ability and behavior performance, we adopted a rodent stop signal task [38] and trained rats under its Go/NoGo mode to carry out the investigation. In our study, the lengthy training duration might promote habits to form. Thus, we included a reversal version of the Go/NoGo task, through which we intended to decipher whether the behavioral flexibility was affected by habits.

## Materials and methods

### Animals

Forty-six male and 51 female Sprague–Dawley (SD) rats, which were purchased from a commercial supplier (Shanghai SLAC Laboratory Animal Co. Ltd, Shanghai, China), were used. All rats were housed in a temperature- (24 ± 2 °C) and humidity- (55 ± 15%) controlled vivarium with a 12-h light/dark cycle (lights on: 8:00 am–8:00 pm; light intensity: 30 Lux). The rats had access to food and water ad libitum and were housed in a group of 3–5 per cage according to their body weights. They were allowed to acclimate the research facility for a minimum of 7 days before the experiments. The training usually started after a minimum of 2-day handling when the rats were over 100 g. Once the training started, the water consumption of individual rat was limited to 15–20 ml per day and provided at various time after each training session. The rats were allowed for an additional 2-h free water access during the training-off period. The body weight of each rats was monitored regularly. If the body weight reduced 20% from the standard SD rat growing chart, the rats were provided with additional water till the body weight recovered. The experimental protocol in this study was approved by the Institutional Animal Care and Use Committee of Shanghai Jiao Tong University.

### Behavioral apparatus

**Fig. 1:**
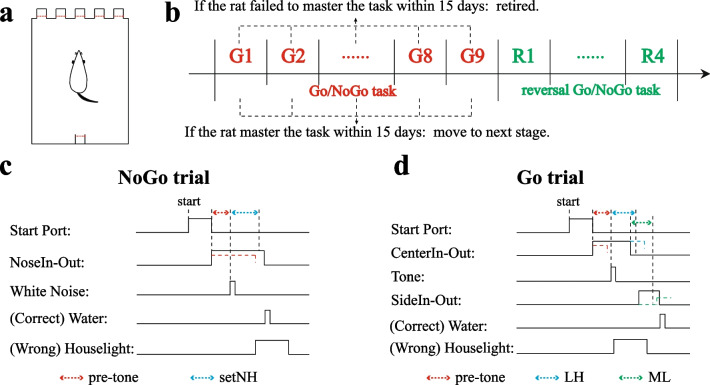
Rodent Go/NoGo task protocols. **a** Illustration of the behavioral apparatus. Five nose ports and one reward delivery port are located in two narrow walls of the apparatus with inferred beam emitters and detectors installed inside. **b** Flowchart of the training processes. The regular Go/NoGo task consists of nine stages, where only NoGo trials are available in G1 and Go trials are introduced in G2. The proportion of NoGo and Go trials is adjusted through stages G1–9. The reversal Go/NoGo task consists of four stages. For regular Go/NoGo task, the learning phase of each stage is set to 15 days for maximum and the rats will be removed for further analysis when beyond the limit. After the rats reach the setting success criteria, varied consolidation phases are applied to individual G stage and R stage. **c** NoGo trial protocol. When the start port lit up, rats are allowed to poke in freely. The pre-tone time starts counting down once poking behavior was detected. After the white noise cue, rats are required to hold in place for certain period. Failure of holding will induce 8 s house light punishment instead of water reward. **d** Go trial protocol. Rats can poke into the start port freely after it lit up. One of the Go cues will be played upon the end of pre-tone delay. Rats are required to pull noses out within a limited time (LH), then poke into the corresponding side port within the setting time limit (ML). Successful performance will trigger a drop of reward water to be delivered

The behavioral apparatuses were custom-built with a dimension of 35(w) × 25(d) × 34(h) cm (Fig. 1a). On one of the narrow side, there were five square nose ports (2.5 cm) with a center-to-center distance of 5 cm. Inside each nose port, the inferred beam emitter and detector were installed to detect rats’ poking behavior. Opposite to the nose port side, a similar port was installed for reward delivery. A speaker was installed on the upper right corner above the reward port for playing the acoustic cues and a 20-W house light was installed above the top of the apparatus for punishment delivery. The behavioral apparatus was controlled by an Arduino board (Mega 2560, Turin, Italy) with custom-written software. The individual behavioral apparatus was enclosed within a dark sound-proof box and cleaned regularly.

### Behavioral task

A rodent Go/NoGo task was used in current study (Fig. 1c, d). The task was composed of three trial types: left Go trial (directed by a 2-kHz tone and requiring the rats to move its nose from the current nose port to the left adjacent nose port), right Go trial (directed by a 6-kHz tone and requiring the rats to move its nose from the current nose port to the right adjacent nose port) and NoGo trial (cued by white noise and requiring the rats to stay in the current nose port for not less than the setting NoGo holding (setNH) time). All the auditory cues had a duration of 50 ms and a decibel level of 80 dB. At the beginning of each trial, one of the three center nose ports was lit randomly. The rat was required to poke into it and hold still for a varied period (pre-tone duration). Once a cue was played, the rat needs to make proper actions accordingly. There were two critical setting time limits which were used to enforce the association between the cues and rats’ actions. The limited holding time (LH), within which the rat had to pull its nose out from the previously entered port after the onset of the Go cues; and the movement limit time (ML), within which the rat had to poke into one of the adjacent ports after withdrawing from the previously entered port. A drop of sterile water (~ 20 µl), which was triggered to be delivered from a stainless steel tube inside the reward port, served as reward for the correct performance. The next trial would only be started when the rat retrieved the reward. On the contrary, the house light would be turned on for 8 s after incorrect trials. The inter-trial interval was randomly generated between 500 and 1500 ms.

### Training strategy

The rats were trained one session per day for 6 days each week. All the rats were trained in the fixed time during the day with the designated training apparatus. Each training sessions lasted for 90 min. The timeline of the training procedure is illustrated in Fig. 1b. Training starts with regular Go/NoGo task (G stage), in which NoGo trial and Go trial were introduced to rats in stage G1 and G2, respectively, in order to facilitate the learning process. Therefore, we considered stage G1 and G2 as initial task learning periods. All other G stages were considered as performance optimization periods. After stage G9, we performed a reversal version of Go/NoGo task (R stage) for a subset of rats (male: 15; female: 9), where the meanings of Go cues were swapped. The detailed setting parameters for each stage are shown in Table 1.

**Table 1:** Setting parameters for each training stage

Stage	PreT (ms)	LH (ms)	ML (ms)	setNH (ms)	Rats # M/F	Trial ratio Go:NoGo	Success criteria		Consolidation days
							Go%	NoGo%	
G1	10–1500	2000	2000	0–400	46/51	0:1	N/A	70	4
G2	50–100	2000	2000	400	43/49	2:3	55	60	4
G3	50–100	2000	2000	400	40/47	1:1	70	70	2
G4	80–300	1500	1500	450	36/40	1:1	70	70	2
G5	150–500	1300	1300	500	34/36	1:1	70	70	2
G6	200–800	1000	1000	550	32/32	1:1	70	70	2
G7	400–1000	800	800	600	28/32	1:1	70	70	2
G8	500–1200	700	700	600	25/28	2:1	70	70	2
G9	500–1500	600	600	600	25/26	2:1	70	70	6
R1	500–1500	600	600	600	15/9	2:1	70	70	2–4
R2	500–1500	600	600	600	11/7	2:1	70	70	2–4
R3	500–1500	600	600	600	11/7	2:1	70	70	2–4
R4	500–1500	600	600	600	10/7	2:1	70	70	2–4

G1–G9: regular Go/NoGo task stages; R1–R4: reversal Go/NoGo task stages. **PreT:** a randomly generated delay time between center nose port entry and audio cue onset; specific PreT ranges were shown in the table for each stage. For stage G1, the initial PreT range was set to 10–50 ms, the lower and upper limits were increased for 10 ms and 20 ms, respectively, after each correct trial till the range reached to 500–1500 ms. **LH:** limited holding time, the time limit within which the rats were allowed to stay in the entered center port after Go cues. **ML:** movement limit time, the time limit within which the rats had to enter one of the adjacent side ports after pulling out from the center port in the Go trial. **setNH:** setting NoGo holding time, the minimal time required the rats to hold still in the entered center port in the NoGo trials after the audio cue. For stage G1, the initial setNH was 0 and increased for 5 ms after each correct trial before reaching 400 ms. **Rats #:** the number of rats for male and female in each stage. **Trial ratio:** the ratio of Go and NoGo trials in each stage. For stage G1, the session contained only NoGo trials. **Success criteria:** the empirical setting criteria (correct rate for Go and NoGo trials within session) to judge the rats mastered each stage. **Consolidation days:** additional training length after the rat reached the setting success criteria

There were three critical phases for each stage, of which the rat did not meet the requirements would be removed from the following stage analysis. The session would be considered as valid when there were a minimum of 150 trials except for the learning phase of the stage G1:

1. Learning phase
  The rats mastered the core requirements through trial and error when they were exposed to the Go/NoGo task for the first time. In addition, they need time to gradually adapt to the changing setting parameters which force them to react faster. We allowed the rats to learn each stage for a maximum of 15 days in order to minimize the age influence on the learning and performance [39, 40]. If a rat failed to master the task within this limit, further analysis would exclude it. However, we did not enforce this 15-day limit for the reversal Go/NoGo task since its main purpose was to study the adjustment for habitual actions.
2. The day for mastering each stage
  Along the forwarding of the learning phases, the rats’ performance would be improved. When the correct rates for both Go and NoGo trials reached our setting goals for a given stage, we considered that the rats mastered that stage and were eligible to be included in the next stage. The required staged correct rates for each trial type are listed in Table 1.
3. Consolidation phase
  After the rats mastered a given stage, we continued the training with the same setting parameters for additional duration to consolidate rats’ performance before advancing them into next stage. The duration of consolidation for each stage is described in Table 1.

### Data analysis

All behavioral performance data were recorded in PC. The data analysis was performed with custom-written Matlab scripts and GraphPad Prism (GraphPad Software, La Jolla, CA, USA). Multiple statistical tests, including Student’s *t*-test, Chi-square test, Wilcoxon signed rank test and two-way ANOVA with repeated measures, were used for different comparisons. For all the cumulative distribution results, the effect size was determined by a standardized mean difference through Cohen’s *d* formula. A linear regression model with categorical covariates was used to estimate the relationship between trials per session and reaction time or movement time.

## Results

### Sex influences on the regular Go/NoGo task performance

**Fig. 2:**
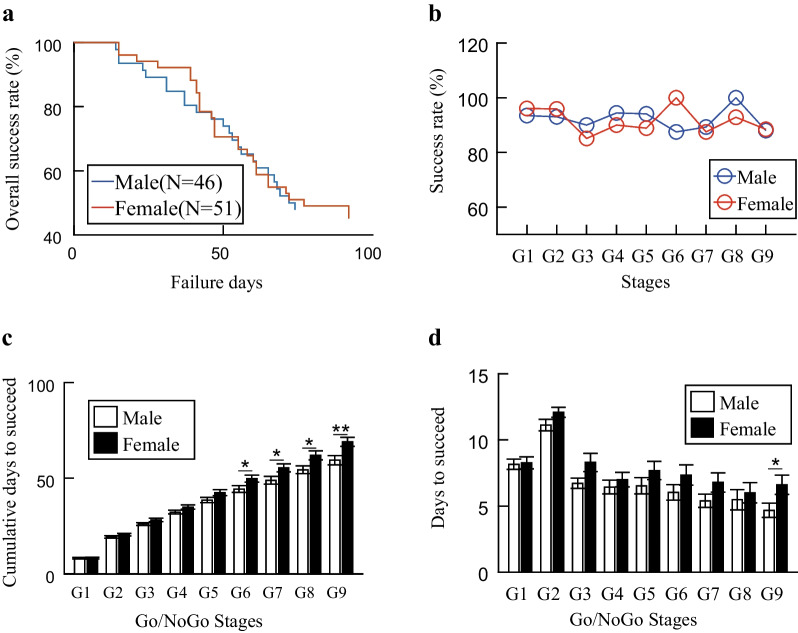
Performances for regular Go/NoGo task. **a** Overall success rate as a function of progression time for male and female rats. The success rates were calculated relative to the initial number of rats from the beginning of stage G1, whenever there was rat failed to meet the required criteria. The success rates for male and female rats were similar (Wilcoxon rank sum test, *p* = 0.77). **b** Success rates for individual stage. The success rates were calculated for each stage based on the total number of animals remaining for that stage. No significant differences were detected between male and female rats (paired *t*-test, *p* = 0.79). **c** Accumulating time needed for mastering each stage (mean ± SEM). No statistically significant differences were detected for stages G1–G5. The female rats need longer time from stage G6 through stage G9 (Student’s *t*-test, pG6 = 0.04, pG7 = 0.03, pG8 = 0.02 and pG9 = 0.006). **d** Time needed for mastering individual stages (mean ± SEM). No statistically significant differences were detected for stages G1–G8. The female rats needed longer time to master stage G9 than the male rats (Student’s *t*-test, *p* = 0.04)

To investigate the effect of sex on learning of the regular Go/NoGo task, we first compared the overall success rates between male and female rats. No statistically significant difference was detected (Fig. 2a; *p* = *0.77, Wilcoxon rank sum test*). With the progression of training, some rats would be removed due to failure to meet our setting requirements, which caused reduction of animal numbers after each stage. Therefore, we calculated the success rate for each stage based on the actual rats’ number of that stage, respectively. The statistical test indicated that both male and female rats had similar success rates throughout all stages (Fig. 2b; *p* = *0.79, paired t-test*). The success rate is only a general performance index, however, cannot reflect the progression in performing the task. We wondered whether it took similar duration for them to reach the staged task-standards. Then, we extracted the accumulating time spent for mastering each stage, our results indicated that it took more time for the female rats in stages G6 ~ G9 (Fig. 2c; *pG6* = *0.04, pG7* = *0.03, pG8* = *0.02 and pG9* = *0.006, Student’s t-test*). The accumulating time for mastering each stage showed the total time needed to master a given stage from the initial training, which made a bit harder to visualize the actual time needed to master each stage. Therefore, we extracted the actual time spent for mastering each stage, the results revealed that the female rats needed more time in stage G9 (Fig. 2d, p = *0.04, Student’s t-test*). Although the actual time needed for each stage did not reach significance for stage G1 ~ G8, the female rats needed more time to master each stage than the male rats (Fig. 2d). Interestingly, the trivial time differences reached significance in stage G6 and thereafter when presented in an accumulating manner (Fig. 2c). These results demonstrated that although little difference can be observed for the proportion of rats who mastered the Go/NoGo task, the female rats required more time for optimizing their performance.

Trial and error was the unique strategy, through which the rats learned to perform the task. In the initial learning period (G1 and G2), both trial types were introduced to the rats for the first time and the rats were required to learn the new rules. In the performance optimization period (G3–G9), however, the rats learned to confine their performance within our stage-specific settings. After comparing the performances for each stage, we observed similar results; therefore, we combined stages G3–G9 data together in all following analysis.

**Fig. 3:**
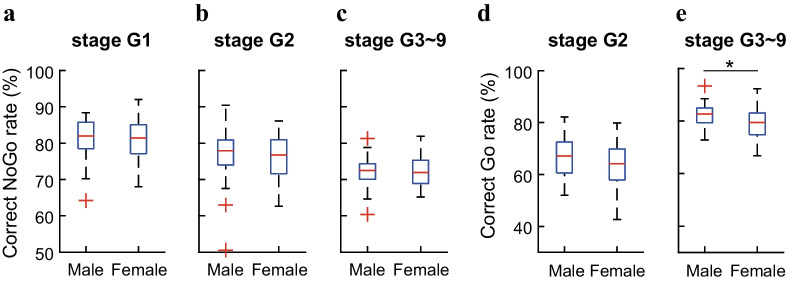
Correct performance rates for Go and NoGo trials of all successful rats in different regular stages. **a** Correct NoGo rates in stage G1, the male and female rats displayed similar performances (*p* = 0.92). **b** Correct NoGo rates in stage G2, the male rats exhibited little difference from the female rats (*p* = 0.40). **c** Correct NoGo rates in stages G3–G9, no difference existed between male and female rats (*p* = 0.90). **d** Correct Go rate in stage G2, no statistically significant difference between male and female rats (*p* = 0.10). **e** Correct Go rate in stages G3–G9, the male rats had higher correct Go rate than the female rats (Student’s *t*-test, *p* = 0.01). Student’s t-tests were used for significance level detection. The length of whiskers was set to 1.5 times of Interquartile ranges, respectively. Automatically detected data outliers were marked as red cross

In the learning phases for all stages, the rats were exposed to either a new trial type or an increased difficulty level. The performance was improved progressively. On the contrary, the rats’ performance should be more stable in the consolidation phases since they passed the requirements for given stages, which reflected the rats’ ability for executing the task. We did not observe any difference for the total trials needed to master any given stage between male and female rats. Therefore, we further compared the correct rates during the consolidation phases for all stages. Our results revealed that the correct rates for NoGo trials were similar between male and female rats for all stages (Fig. 3a–c). For the correct rates of Go trials, the male rats were slightly higher than the female rats without statistically significant difference in stage G2 (Fig. 3d). However, the male rats gained higher correct Go rate than the female rats in stages G3–G9 (Fig. 3e; *p* = *0.01, Student’s t-test*). If taking the interquartile range as measurement of data variability, we could observe less variations for the both correct NoGo and Go rates in male rats from Fig. 3, thus, our data implied that the male rats displayed better performance stability than the female rats.

### Female rats took more cautious strategy

**Fig. 4:**
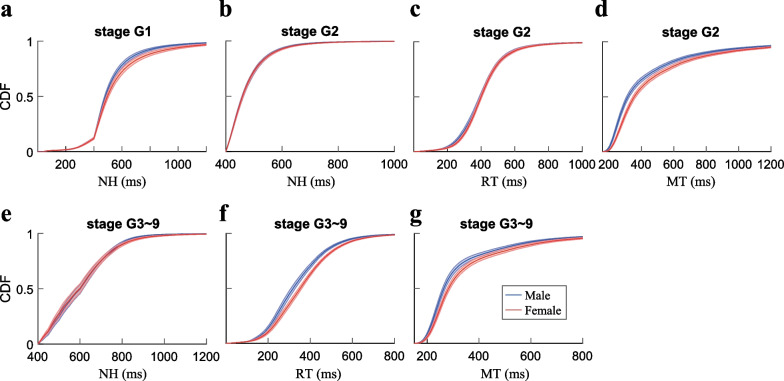
Actual NoGo holding time, reaction time and movement time in different regular stages. **a** The cumulative distribution of NH time in stage G1. The NH time was shorter for male rats than female rats with an effect size of − 0.34 (*p* = 2.22 × 10^–158^). **b** The cumulative distribution of NH time in stage G2. The statistical test revealed a significant difference between male and female rats with an effect size of 0.02 (*p* = 4.03 × 10^–10^). **c** The cumulative distribution of RTs in stage G2. The RTs of male rats were shorter than female rats with an effect size of − 0.14 (*p* = 3.17 × 10^–162^). **d** The cumulative distribution of MTs in stage G2. The MTs of male rats were shorter than the female rats with an effect size of − 0.63 (*p* = 6.85 × 10^–172^). **e** The cumulative distribution of NH time in stages G3–9. The NH time of the male rats was shorter in the later part than the female rats with an effect size of 0.05 (*p* = 2.75 × 10^–5^). **f** The cumulative distribution of RTs in stage G3–9. The RTs of male rats were shorter than the female rats with an effect size of − 0.56 (*p* = 7.57 × 10^–125^). **g** The cumulative distribution of MTs in stage G3–9. The MTs of female rats were longer than the male rats with an effect size of − 0.41 (*p* = 2.84 × 10^–108^). Wilcoxon signed rank tests were used for significance level detection

The performance quality is closely related to the quantity of reward which serves as a motivational drive. Different behavioral strategies are required for performing different trial types. Stage G1 only contains NoGo trials. In the beginning of the trial, one of five nose ports would be lit up, the rats were required to hold in the entered nose port after the audio cue for a minimal duration according to the experimental settings, which was the key to succeed in NoGo trials. In order to facilitate the learning process, the setting NoGo holding time (setNH) was increased for 5 ms after every correct trial and fixed at 400 ms. For the cumulative distribution of actual NoGo holding (NH) time illustrated in Fig. 4a, our data indicated that there were similar distributions for male and female rats when the setNHs were below 400 ms in the early portion of individual sessions. Upon the setNHs were fixed at 400 ms, however, the male rats held significantly shorter than the female rats (Fig. 4a; *p* = *2.22* × *10*^*–158*^* with an effect size of *− *0.34, Wilcoxon signed rank test*).

In stage G2, the rats well-learned the NoGo trial, then we fixed the setNHs to 400 ms. Meanwhile, we introduced Go trials to the rats in a subset of trials (40%). At this stage, we observed significant difference for the actual NH time between male and female rats with a much smaller effect size (Fig. 4b; *p* = *4.03* × *10*^*–10*^* with an effect size of 0.02, Wilcoxon signed rank test*). For the newly introduced Go trials, the rats had to establish the association between the Go cues and the corresponding side ports. After the onset of Go cues, the rats were required to pull out from the center nose port, where we defined the duration between the Go cue onset and the nose exit as reaction time (RT) to the Go cues. Next, the rats were required to enter the correct adjacent side ports directed by the Go cues. The time from exit of center port to the entry of the target ports was defined as movement time (MT). The rats need to complete these two actions promptly within respective time limit (LH and ML) in order to obtain the water reward. Although the RTs cumulative distribution showed slightly separation at early part (Fig. 4c), there was a statistically significant difference exited between male and female rats with a very smaller effect size (*p* = *3.17* × *10*^*–162*^* with an effect size of *− *0.14, Wilcoxon signed rank test*). We further compared the MTs between the two groups of rats, the female rats had significantly longer MTs than the male rats (Fig. 4d; *6.85* × *10*^*–172*^* with an effect size of *− *0.63, Wilcoxon signed rank test*).

In stages G3–G9, there were no new cues to learn, but the task difficulty level increased. The rats were required to adjust their behavioral performance to the staged setting parameters accordingly in order to receive reward. For the NoGo trials, the NH time distribution overlapped for the majority part (Fig. 4e), even though, the NH time distributions started to separate in the later part (> 700 ms), which induced the significant difference for the NH time between male and female rats with much smaller effect size (Fig. 4e; *p* = *2.75* × *10*^*–5*^* with an effect size of 0.05, Wilcoxon signed rank test*). Furthermore, we observed significantly shorter RTs (Fig. 4f; *p* = *7.57* × *10*^*–125*^* with an effect size of *− *0.56, Wilcoxon signed rank test)* and MTs (Fig. 4g; *p* = *2.84* × *10*^*–108*^* with an effect size of *− *0.41, Wilcoxon signed rank test*) distributions for male rats than the female rats.

Our data demonstrated that the female rats tended to hold longer than the male rats when performing the NoGo trials in both initial NoGo stage (G1) and the later performance optimization stages (G3–G9). Furthermore, the female rats displayed slower RTs exclusively in stages G3–G9 and longer MTs for all Go trials (stages G2 and G3–9). Taken together, it seems that the female rats might take more cautious strategies in order to achieve success in the regular Go/NoGo task since they generally reacted to cues and completed the trials at a slower pace.

### Biased performance induced failure of reaching the success criteria

**Fig. 5:**
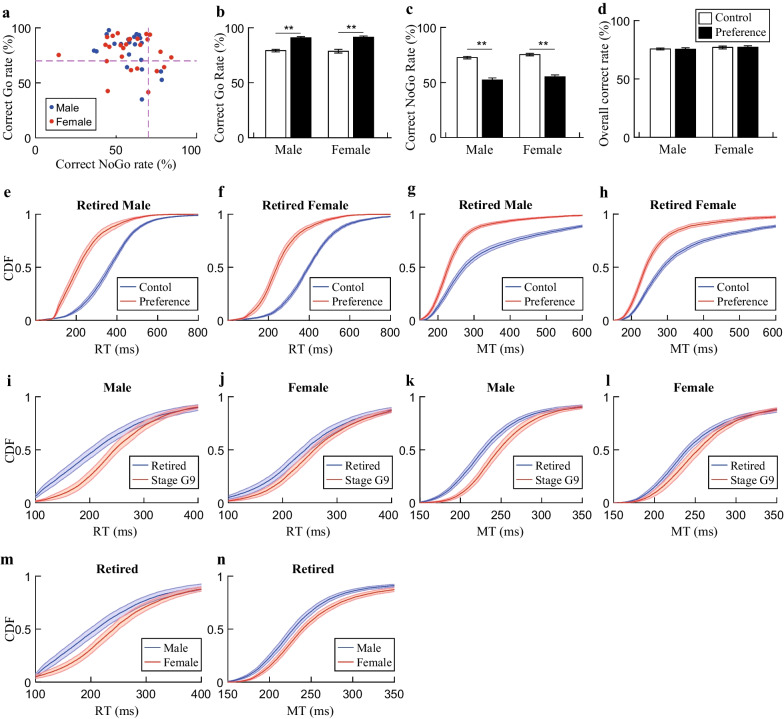
Overall performances for retired rats. **a** Plot of correct Go rates as a function of correct NoGo rates for all retired rats (N_male_ = 21; N_female_ = 26). **b** Comparisons of the correct Go rates before and after the Go-preference developed for the retired rats (mean ± SEM). Both retired male and female rats exhibited significantly higher correct Go rates after Go-preference developed (pMale = 1.29 × 10^–7^ and pFemale = 1.39 × 10^–6^). **c** Comparisons of the correct NoGo rates before and after the Go-preference developed for the retired rats (mean ± SEM). Both retired male and female rats exhibited significantly lower correct NoGo rates after Go-preference developed (pMale = 5.74 × 10^–10^ and pFemale = 2.62 × 10^–10^). **d** Comparisons of overall correct rates for the retired rats before and after the Go-preference developed (mean ± SEM). No significant difference was observed for both retired male and female rats (pMale = 0.92 and pFemale = 0.93). **e**, **f** Cumulative distribution of RTs before and after the Go-preference developed for retired rats. The RTs were significantly shortened after Go-preference developed for male (*p* = 7.96 × 10^–127^ with an effect size of 1.97) and female (*p* = 1.47 × 10^–132^ with an effect size of 2.41) rats, respectively. **g**–**h** Cumulative distribution of MTs before and after the Go-preference developed for retired rats. The MTs were significantly shortened after Go-preference developed for male (*p* = 1.27 × 10^–75^ with an effect size of 1.04) and female (*p* = 1.43 × 10^–75^ with an effect size of 1.38) rats, respectively. **i**–**j** Comparison of cumulative distributions of RTs of retired rats with Go-preference and rats in stage G9. The RTs were significantly shortened for Go-preference retired male rats (*p* = 3.62 × 10^–47^ with an effect size of − 0.55) and female rats (*p* = 4.17 × 10^–51^ with an effect size of − 0.22) than the rats in stage G9. **k**–**l** Comparison of cumulative distributions of MTs for retired rats with Go-preference and rats in stage G9. The MTs were significantly shortened for Go-preference retired male rats (*p* = 9.85 × 10^–35^ with an effect size of − 0.80) and female rats (*p* = 1.73 × 10^–29^ with an effect size of − 0.54) than the rats in stage G9. **m** Comparison of cumulative distributions of RTs of retired male and female rats with Go-preference. The RTs were significantly shortened for retired male rats than retired female rats (*p* = 4.17 × 10^–51^ with an effect size of − 0.43). **n** Comparison of cumulative distributions of MTs of retired male and female rats with Go-preference. The MTs were significantly shortened for retired male rats than retired female rats (*p* = 9.85 × 10^–35^ with an effect size of − 0.40). Two-way ANOVA with repeated measures and post hoc Holm–Sidak tests were used for **b**–**d**. Wilcoxon signed rank test was used for **e**–**n**. For Fig. **b**–**n**, N_male_ = 15 and N_female_ = 14

In order to evaluate the task performance quality, the success criteria for each stage were set empirically (Table 1). When the rats failed to reach certain stage-specific criteria after the maximal training length (15 days), they were retired from the staged training. The retired rats, thereafter, were trained continuously on their own pace and some of them adapted to the adjustment of parameters without any problem. We were curious, however, about the causes for the failure to meet our setting criteria. Then, we averaged the correct rates for the last three training sessions, which should be the optimized final performance status, for all retired rats in stages G2–G9 and plotted the correct Go rates as a function of the correct NoGo rates. Our results revealed that 76% male and 62% female retired rats developed preference to perform Go trials, where the retiring rates were similar between male and female rats (Fig. 5a; χ^*2*^*(1, N* = *47)* = *1.15, p* = *0.28, Chi-square test*). Since all the retired rats have succeeded at certain stage before retirement, we next selected three sessions in the consolidation phase before retiring as control sessions, and three sessions close to the end of training as preference sessions. We then performed two-way ANOVA with repeated measures, where sex as the independent factor and session as the repeated factor. The statistical analysis revealed no sex by session interactions for both Go (*F*_*(1.27)*_ = *0.23, p* = *0.63*) and NoGo trials (*F*_*(1.27)*_ = *0.003, p* = *0.95*). However, post hoc comparison with Holm–Sidak test revealed that both male and female retired rats exhibited significantly higher correct Go rates (Fig. 5b; *pMale* = *1.29* × *10*^*–7*^* and pFemale* = *1.39* × *10*^*–6*^*, two-way ANOVA with repeated measures and *post hoc* Holm–Sidak test)* and lower correct NoGo rates (Fig. 5c; *pMale* = *5.74* × *10*^*–10*^* and pFemale* = *2.62* × *10*^*–10*^*, two-way ANOVA with repeated measures and *post hoc* Holm–Sidak test*) after retirement. In our current behavioral paradigm, the water reward served as an incentive drive, any performance failure would reduce the total reward obtained per session. We were curious whether the total rewards obtained within session would be affected after the retired rats developed this kind of biased performance behavior. Therefore, we compared the overall correct rates for the control and preference sessions. There was no sex by session interaction observed *(F*_*(1,27)*_ = *0.02, p* = *0.88*), and little influence was observed for both retired male and female rats as well (Fig. 5d; *pMale* = *0.92 and pFemale* = *0.93, two-way ANOVA with repeated measures and *post hoc* Holm–Sidak test*). Our results indicated that all the retired rats could still retrieve rewards for over 70% of total trials per session after they developed preference to perform Go trials.

Once the rats preferred to perform Go trials, it is reasonable to speculate that they tended to react to the Go cues faster. Not surprisingly, the cumulative distributions showed significantly shorter RTs and MTs for preference sessions of both male and female retired rats than their control sessions with medium-to-large effect sizes, respectively (Fig. 5e–h). Since the retired rats had undergone similar training durations as the rats that completed all regular Go/NoGo stages, the performance of both groups of rats should reach a stable plateau, respectively. We then compared the performance between the retired rats and the rats in the consolidation phase of stage G9. Our analysis revealed that the retired rats exhibited significantly shorter RTs in their preference sessions than the RTs of the rats in stage G9 for both male and female rats, where the difference was much bigger among male rats (Fig. 5i, j, m). Meanwhile, the retired rats displayed significantly shorter MTs than that of the rats in stage G9 (Fig. 5k, l), while the male rats possessed bigger difference than the female rats (Fig. 5n). Our speculations were validated. The above results demonstrated that the rats used a Go–NoGo trade-off strategy, through which they could maintain minimal influences on the overall rewards by focusing on the Go trials.

### Sex influences on the behavioral flexibility

Studies indicated that a great number of repetitions for a certain action would promote habit development [23, 41]. Although it was not tested whether the behavioral responses are habitual or not with any devaluation experimental design, the rats had undergone numerous training sessions when they passed the final Go/NoGo stage (G9) in our current study, in which the training duration was longer than that reported elsewhere for rat habit study [42]. It is logical to ask whether sex is a factor, which influences the adjustment for a habited action. Then, we trained a subset of rats (Table 1), who passed stage G9, for a reversal Go/NoGo task with swapped Go cues.

**Fig. 6:**
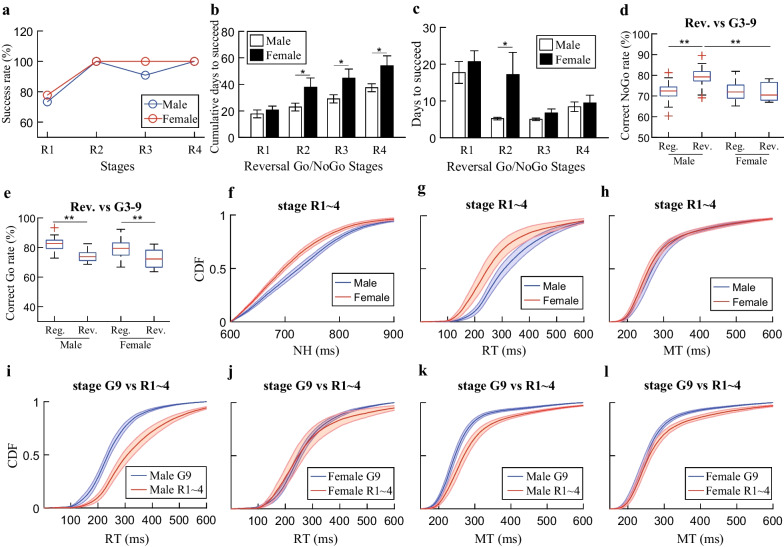
Overall performances for reversal Go/NoGo task. **a** Success rates for individual stage. The success rates were calculated for each reversal stage based on the total number of animals remaining in that stage. No significant differences were detected (Paired *t*-test, *p* = 0.22). **b** Accumulating time needed for mastering each reversal stage (mean ± SEM). No statistically significant differences were detected for stage R1. The female rats need longer time from stages R2–R4 (Student’s *t*-test, *p* = 0.04 for stage R2–R4). **c** Time needed for mastering individual reversal stages (mean ± SEM). No statistically significant differences were detected for stages R1, R3 and R4. The female rats needed longer time to master stage R2 than the male rats (Student’s *t*-test, *p* = 0.02 for stage R2). **d** Comparisons of correct NoGo rates between reversal stages and stages G3–G9. The correct NoGo rates of male rats in the reversal stages were significantly higher than both male rats of stages G3–9 and female rats of Stages R1–R4 (*p* = 1.75 × 10^–5^ and *p* = 0.003, respectively). **e** Comparisons of correct Go rates between reversal stages and stages G3–G9. The correct Go rates of both male and female rats in the reversal stages were significantly decreased than those of stages G3–G9 (*p* = 6.18 × 10^–7^ and *p* = 0.005 for male and female rats, respectively). **f** Comparison of NH time for male and female rats in the reversal stages. The NH time was significantly shorter for the female rats than that of the male rats (*p* = 6.08 × 10^–51^ with an effect size of 1.49). **g**–**h** Comparison of RTs and MTs for male and female rats in the reversal stages. No significant differences were detected for RTs (*p* = 5.98 × 10^–100^ with an effect size of 0.98) and MTs (*p* = 3.56 × 10^–8^ with an effect size of 0.29). **i**–**j** Comparison of RTs for male and female rats between the reversal stages and stage G9. The RTs of reversal stages were significantly longer than that of stage G9 for male (*p* = 6.03 × 10^–100^ with an effect size of − 1.32) and female (*p* = 6.28 × 10^–26^ with an effect size of 0.06) rats, respectively. **k**–**l** Comparison of MTs for male and female rats between the reversal stages and stage G9. The MTs of reversal stages were significantly longer than that of stage G9 for male (*p* = 1.27 × 10^–75^ with an effect size of − 0.86) and female (*p* = 1.27 × 10^–75^ with an effect size of − 0.41) rats, respectively. Two-way ANOVA with repeated measures and post hoc Holm–Sidak tests were used for **d** ~ **e**. Wilcoxon signed rank test was used for **f**–**l**

Our data revealed that the proportion, of which the rats passed each reversal stage, did not show any significant difference between male and female rats (Fig. 6a). In the first stage of reversal Go/NoGo task (R1), we observed similar training durations for them to switch the association of Go cues (Fig. 6b). However, the female rats need significantly longer time than the male rats to master stages R2–R4 from the initial reversal stage (Fig. 6b; *p* = *0.04, Student’s t-test*). If we compared the individual time needed for mastering each reversal stage, female rats needed significantly longer time in stage R2 (Fig. 6c, p = *0.02, Student’s t-test*), though there was a trend which female rats needed longer time.

Similar as seen in stages G3–G9 of the regular Go/NoGo task, the performance of each reversal Go/NoGo stage was similar. Therefore, we combined stages R1–R4 together and analyzed the data during all consolidation phases. We then performed two-way ANOVA with repeated measures, where sex was the independent factor and stage was the repeated factor, for correct rates from stage R1–R4 and stage G3–G9. For NoGo trials, there was a strong sex by stage interaction (Fig. 6d; *F*_*(1,92)*_ = *9.18, p* = *0.003*). The interaction effect is clearly shown in Fig. 6d, where the male rats had significantly higher correct NoGo rate in stage R1–R4 than that of stage G3–G9 (*p* = *1.75* × *10*^*–5*^, *two-way ANOVA with repeated measures and *post hoc* Holm–Sidak test*). In addition, the correct NoGo rate was significantly higher for male rats than female rats in stage R1–R4 (*p* = *0.003, two-way ANOVA with repeated measures and *post hoc* Holm–Sidak test*). For Go trials, no sex by stage interaction was detected (Fig. 6e; *F*_*(1,92)*_ = *0.32, p* = *0.57*). However, post hoc comparison using Holm–Sidak test revealed that the correct rates of both male and female rats decreased significantly in stage R1–R4 than those of stage G3–G9. However, no significant difference was detected between male and female rats in stage R1–R4 (*p* = *0.76, two-way ANOVA with repeated measures and *post hoc* Holm–Sidak test*).

Since there was a strong sex by stage interaction for NoGo trials and significantly decreasing for Go correct rates, we then made comparisons for NH time, RTs and MTs within stage R1–R4 and with those of stage G9 for male and female rats. The results showed that the female rats had shorter NH time than that of male rats in stage R1–R4 (Fig. 6f; *p* = *6.08* × *10*^*–51*^* with an effect size of 1.49, Wilcoxon signed rank test*). This is as expected since the female rats had lower correct NoGo rate. Next, we plotted the RTs distributions, the male rats had significantly shorter RTs than the female rats in stage R1–R4 (Fig. 6g; *p* = *5.98* × *10*^*–100*^* with an effect size of 0.98, Wilcoxon signed rank test*). When the comparisons were made with stage G9 which represented the final stable performance status in regular Go/NoGo task, however, significant differences were detected for both male and female rats. The male rats had clearly longer RTs in stage R1–R4 than that of stage G9 (Fig. 6i; *p* = *6.03* × *10*^*–100*^* with an effect size of *− *1.32, Wilcoxon signed rank test*), while intermingled results were observed for female rats with shorter RT in the faster portion and longer RTs in the slower portion for stage R1–R4 than that of stage G9 (Fig. 6j; *p* = *6.28* × *10*^*–26*^* with an effect size of 0.06, Wilcoxon signed rank test*). In the last comparison made for MTs, we observed similar MTs distribution in stage R1–R4 between male and female rats (Fig. 6h; *p* = *3.56* × *10*^*–8*^* with an effect size of 0.29, Wilcoxon signed rank test*), where the majority of distributions were overlapped. However, both male and female rats exhibited significantly longer MTs in stage R1–R4 than that of stage G9 (Fig. 6k, l; *p* = *1.27* × *10*^*–75*^* with an effect size of *− *0.86 and p* = *1.27* × *10*^*–75*^* with an effect size of *− *0.41, respectively, Wilcoxon signed rank test*).

This part of data demonstrated that both male and female rats could adapt the reversed experimental conditions in the confined time frames, however, the influence on the performance for both male and female rats seems complicated.

## Discussion

Go/NoGo tasks are widely used to probe neural mechanisms for executive function and response inhibition [43–45]. Pre-clinical studies require more aggressive investigations with animal models. The lengthy training durations, however, are main drawbacks for available rodent Go/NoGo tasks [46, 47]. After adopting the behavioral paradigm, therefore, critical modifications were made with pre-defined stage-specific parameters (Table 1), which greatly facilitated the training process. In addition, the data comparability was enhanced as well. After the initial acquisition periods, the rest training sessions are mainly for behavioral optimization according to the stage-specific parameters, through which stereotyped performances tend to be developed [23]. Consequently, an opportunity was created to investigate the behavioral flexibility after potential habits formation.

The influences of sex on learning and memory have been extensively investigated [48–50]. Inconsistent findings were reported [4, 6, 18, 51], which is likely induced by task-specific attributions. The learning ability can be expressed through various indices. In our behavioral tasks, we found nearly little sex influences on the general learning ability, except for the slight differences observed in stages G9 and R2 (Figs. 2a, b, 6a). Certain progressive learning information might be concealed, if we solely valued the success rates. Therefore, we performed comparisons between the staged learning durations, which revealed that the male rats outperformed the female rats starting from certain stages (Figs. 2c, 6b). The time needed to master the staged tasks is apparently associated with task difficulty levels. Subtle differences of the time needed to pass each stage would be amplified with continuous accumulations. In addition, a possible cause might be that the male and female rats used distinct dimensions of information to decide what to do [52]. Moreover, the male and female rats might possess different ability to resist the interference between Go and NoGo cues [53, 54].

A series of information processing are required when performing the Go trials [46, 55]. The rats would only initiate the directional movement after the information processing for Go cues in the motor control neural circuits, in turn, which determines the length of RTs to Go cues. Although the statistical test revealed significant differences for the accumulating distribution of RTs in the initial stage (Fig. 4c), the effect size was − 0.14 (*Cohen’s d* method), which indicated that the significance was very limited. This demonstrated similar neural processing for Go cues between male and female rats in the initial stage. In the behavioral optimization stages (G3–G9), apart from the steadily enhanced performance (Fig. 3d, e), the differences of RTs distribution were manifested with increased effect size (Fig. 4f, effect size = − 0.56*, Cohen’s d method*). Ample studies indicated that the females are more cautious than the males in many aspects [56–58]. It is reasonable to deduce, therefore, that the female rats might take more cautious considerations upon increased task difficulty levels, which resulted in longer RTs than that of the male rats. The movement actions, which triggered by the Go cues, need to be maintained till reaching the target port in order to conclude the Go trials. The male rats were consistently observed to finalize the Go trials within a shorter time window (Fig. 4d, g) in regular Go/NoGo task. Either sex-featured movement speed or cautious degree might be contributed to the differences among MTs. Taken together, our results demonstrated that the male rats outweighed the female rats in Go trial performance, which is consistent with previous studies that the males outperformed the females in a variety of spatial tasks [59–61]. The hippocampus displayed different plasticity efficacy between males and females [51, 62]. In addition, numerous studies indicate that the sex differences existed in the prefrontal cortex dependent cognitive functions [63–65]. Therefore, the underlying mechanism might be jointly contributed by the hippocampus and the prefrontal cortex.

Although no movement actions are required in NoGo trials, the information processing for NoGo trials is complicated as well. Two obvious strategies are available to rats with free choice in order to fulfill the NoGo trials. One strategy is to wait much longer than the required time, which will sacrifice the session performance turnover. The other strategy is to estimate the elapsing time internally according to the setNHs, which may induce a higher error rate. We observed little difference for performance (Fig. 3a–c), which indicated a dissociation with the chosen strategies. Although the NH time was significantly different in all stages, the male rats had shorter NH time in stage G1 with a relative bigger effect size of − 0.34. With similar NH time distribution ranges, our NoGo performance data were consistent with previous researches, which indicated that the males were more accurate than the females in estimating time [66–68]. Through estimating the elapsing time, the male rats participated in the task more actively, which might gain more control for the performance pace.

Subjects employ various behavioral strategies according to the task features [69–72]. The learning outcomes vary as well. Hence, it is necessary to perform evaluations after purposeful learnings, through which the learning outcomes can be revealed and adjustments can be applied accordingly. Plentiful studies focused mainly on the performances of who met the experimental setting requirements [73–75]. However, the subgroups, which failed but have learned the task, gained less attention. We found that most retired rats have developed preference to perform Go trials, which is the fundamental cause for the failures (Fig. 5a–c). When this strategy was taken, the immediate question would be whether the total obtained reward was affected. Our data revealed little influence was imposed on the total rewards obtained per session for both retired male and female rats (Fig. 5d). To some extent, the retired rats would rather sacrifice the quantity of reward by focusing on the Go trials, which could potentially speed up the trial cycle and reduce the task difficulty levels. All the retired rats succeeded in certain stages prior to the retirements. With continued training after retirement, the preference should be more pronounced. Since the preference was not an initial feature, we thought that the retired rats should react to the Go cues differently between the periods before and after retirements. Without any surprise, both distributions of RTs and MTs were much shorter for the sessions when retired rats developed preference (Fig. 5e–h), where the male rats exhibited bigger changes relative to their control sessions. In addition, the summations of median RT and median MT were 439 ms and 482 ms for retired male and female rats, respectively. With 400–600 ms of setNHs, it consequently caused the time needed for doing Go trials was less than that of doing NoGo trials for the majority of sessions. The trial cycle would be speeded up and task difficulty levels would be lowered, therefore, with preference development for Go trials, which validated our speculations. When the rats passed the stage G9, it concluded the entire regular Go/NoGo training. Since we continuously trained the retired rats in parallel, all the rats should be well-trained and their performance should be stabilized perfectly. We then wonder whether the Go trial performances were similar between the retired rats and the rats in the consolidation phase of stage G9 at this time point. Further analysis revealed that all the comparisons for RTs and MTs distributions reached significance (Fig. 5i–n). Although age exerted significant influences on behavioral performance [39, 40, 76], our training strategy confined the relative age in similar ranges within each stage, which reduced the age influence to minimum. Unlike humans, neither verbal corrections could be made, nor rats knew their performance deviated. Our data, therefore, reflected the natural strategy choices and evolutions for behavioral performances. We observed two distinctive strategies, “Cue-loyalty” and “Go-preference”, were adopted naturally in performing the regular Go/NoGo task. Although biased performance strategies are likely to increase overall error rates, the underlying neural coding mechanism for preference developing is worthy for further investigation with current paradigm.

Repeated behaviors might be stereotyped to form habits [24, 77]. Although habited behavior could increase efficiency, the behavioral flexibility, which is vital to adapt changing circumstances, would be reduced. After reversing the Go cues, all rats exhibited similar ability to re-establish the associations (Fig. 6a). Nevertheless, the reversal NoGo performance increased dramatically for the male rats, while little change was observed for the female rats (Fig. 6d). The changes consequently induced significant difference for reversal NoGo performances between male and female rats, however, similar NoGo performances were observed in the regular Go/NoGo task. Little changes were made to NoGo trials in the reversal Go/NoGo task, thus, it would be an easy solution, which ensures acquiring similar amount of reward under changing situation, by increasing the success rate for familiar trial type. This hypothesis was further confirmed by longer NH time for male rats (Fig. 6f). It seems that the male rats changed strategy to perform the reversed task in order to maintain the quantity of reward. Moreover, both reversal Go performances were decreased strikingly from regular Go performances (Fig. 6e). Surprisingly, the previous Go performance differences, existed between male and female rats (Fig. 3e), vanished in the reversal stages, which implies more impacts on male rats upon swapped Go cues. In addition, the differences between male and female rats, observed for the distributions of RTs and MTs in the regular Go/NoGo task (Fig. 4f–g), were detectable in the reversal stages as well (Fig. 6g–h). The impact on the performance of male rats was more dramatic, which implies that more stereotyped behaviors might be developed. Therefore, it takes more time for information processing under changed conditions in order to maintain certain level of success, which slowed down the overall Go performance. Reasonably, we thought that the reversed Go cues would decrease RTs and MTs to some extent. The comparisons made between the reversal stages and stage G9 (best performance of all G stages and neighbor stage for R stages), validated our hypothesis (Fig. 6i–l). The potential causes, however, might be different. As discussed previously, the male rats were more resistant to change after the development of habited behavior, which in turn prolonged RTs and MTs. On the contrary, caution is a feature for the females [56–58]; it is not surprising that the female rats took more cautious manners to perform the reversed Go trials, which prolonged the performance duration.

**Fig. 7:**
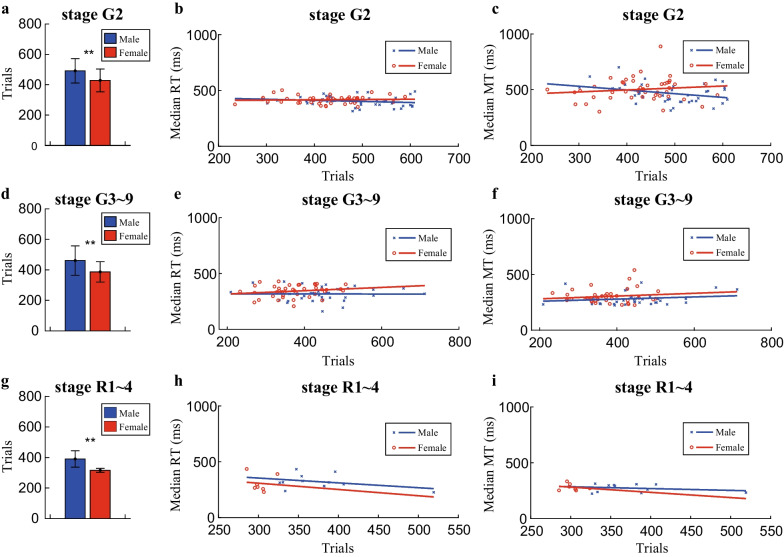
Averaged trials per session and regression model for RT and MT with the averaged trials. **a** Averaged number of trials per session for stage G2 (mean ± SD). The female rats performed significantly less trials in average than the male rats (Student’s *t*-test, *p* = 3.18 × 10^–4^). **b** Linear regression model with categorical covariates was used to test if the RTs can be significantly affected by the number of trials per session in stage G2. The fitted regression model for male rats was: mRT = 449.41–0.10*Trials. The fitted regression model for female rats was: fRT = 405.38 + 0.03*Trials. The overall regression was not statistically significant (*R*^2^ = 0.05, F_(1,81)_ = 1.38 and *p* = 0.25). The slopes of fitted models are similar for male and female rats (F_(1,81)_ = 1.19 and *p* = 0.28). **c** Linear regression model with categorical covariates was used to test if the MTs can be significantly affected by the number of trials per session in stage G2. The fitted regression model for male rats was: mMT = 630.48–0.33*Trials. The fitted regression model for female rats was: fMT = 427.51 + 0.18*Trials. The overall regression was not statistically significant (R^2^ = 0.08, F_(1,81)_ = 2.39 and *p* = 0.05). The slopes of fitted models are not statistically different for male and female rats (F_(1,81)_ = 3.85 and *p* = 0.05). **d** Averaged number of trials per session for stage G3–9 (mean ± SD). The female rats performed significantly less trials in average than the male rats (Student’s *t*-test, *p* = 1.81 × 10^–4^). **e** Linear regression model with categorical covariates was used to test if the RTs can be significantly affected by the number of trials per session in stage G3–9. The fitted regression model for male rats was: mRT = 319.18–0.005*Trials. The fitted regression model for female rats was: fRT = 287.08 + 0.15*Trials. The overall regression was not statistically significant (*R*^2^ = 0.06, F_(1,72)_ = 1.58 and *p* = 0.20). The slopes of fitted models are similar for male and female rats (F_(1,72)_ = 0.81 and *p* = 0.37). **f** Linear regression model with categorical covariates was used to test if the MTs can be significantly affected by the number of trials per session in stage G3–9. The fitted regression model for male rats was: mMT = 239.68 + 0.10*Trials. The fitted regression model for female rats was: fMT = 256.13 + 0.12*Trials. The overall regression was not statistically significant (*R*^2^ = 0.05, F_(1,72)_ = 1.25 and *p* = 0.30). The slopes of fitted models are similar for male and female rats (F_(1,72)_ = 0.02 and *p* = 0.88). **g** Averaged number of trials per session for stage R1–4 (mean ± SD). The female rats performed significantly less trials in average than the male rats (Student’s *t*-test, *p* = 2.71 × 10^–3^). **h** Linear regression model with categorical covariates was used to test if the RTs can be significantly affected by the number of trials per session in stage R1–4. The fitted regression model for male rats was: mRT = 483.21–0.43*Trials. The fitted regression model for female rats was: fRT = 476.09–0.56*Trials. The overall regression was not statistically significant (*R*^2^ = 0.09, F_(1,14)_ = 0.45 and *p* = 0.72). The slopes of fitted models are similar for male and female rats (F_(1,14)_ = 0.002 and *p* = 0.96). **i** Linear regression model with categorical covariates was used to test if the MTs can be significantly affected by the number of trials per session in stage R1–4. The fitted regression model for male rats was: mMT = 334.20–0.16*Trials. The fitted regression model for female rats was: fMT = 422.49–0.47*Trials. The overall regression was not statistically significant (*R*^2^ = 0.07, F_(1,14)_ = 0.33 and *p* = 0.80). The slopes of fitted models are similar for male and female rats (F_(1,14)_ = 0.06 and *p* = 0.81)

Extensive practice is required for mastering a new skill and improving the performance [78, 79]. It is logically disputable that our findings might be biased by the number of trials the rats performed within each session. In order to eliminate this possibility, we have compared the averaged trials per session between male and female rats, then a linear regression model was used to evaluate the correlation of RTs and MTs with the number of trials per session, respectively. Interestingly, the number of trials per session of the female rats was significantly less than that of the male rats in stage G2 (Fig. 7a), stage G3–9 (Fig. 7d) and stage R1–4 (Fig. 7g). However, the female rats exhibited shorter RTs and MTs in the regular Go/NoGo task (Fig. 4c, d, f, g) and longer RTs and MTs in reversal Go/NoGo task (Fig. 6g, h) than those of the male rats, which indicates that the differences in behavioral performance are not related to the number of trials per session. Moreover, the linear regression analyses revealed that the regression model did not reach significance for any of the stages we analyzed (Fig. 7b, c, e, f, h, i). The regression results further confirmed that our observed RTs and MTs from both male and female rats were not dependent on the number of trials per session.

Insightful results were obtained by using Go/NoGo tasks in the clinical studies for self-control [80, 81], which emphasizes the importance of this cognitive paradigm. Sex-related electrophysiological features were identified with Go/NoGo tasks as well [82, 83]. In addition to the findings presented here, our behavioral paradigm may provide a valuable preclinical model for deciphering neural coding and pharmacological mechanisms of sex influences on self-control.

### Perspectives and significance

Our data demonstrated multiple lines of differences between male and female rats in learning and performing the well-designed Go/NoGo tasks. During self-paced learning, different learning or performing strategies might be taken, which might be induced by the function of hippocampus in concert with the prefrontal cortex. Therefore, future studies for neural coding mechanism at the circuits level would enrich our understanding for the sexual dimorphism in learning.

## Data Availability

The datasets used and/or analyzed during the current study are available from the corresponding author on reasonable request.
